# Supplementary material for: Splice-Junction-Based Mapping of Alternative Isoforms in the Human Proteome
Source: Cell Rep. Author manuscript; Available in PMC 2020 Jan 15. (PMC6961840; doi:10.1016/j.celrep.2019.11.026)
Supplement: 2 [file NIHMS1546469-supplement-2.zip › DF1/PXD006675/MitralValve/MitralValve_11_NMRK2_TARPAASQQDSPWWTCTAAGTSMTNGGK.pdf]

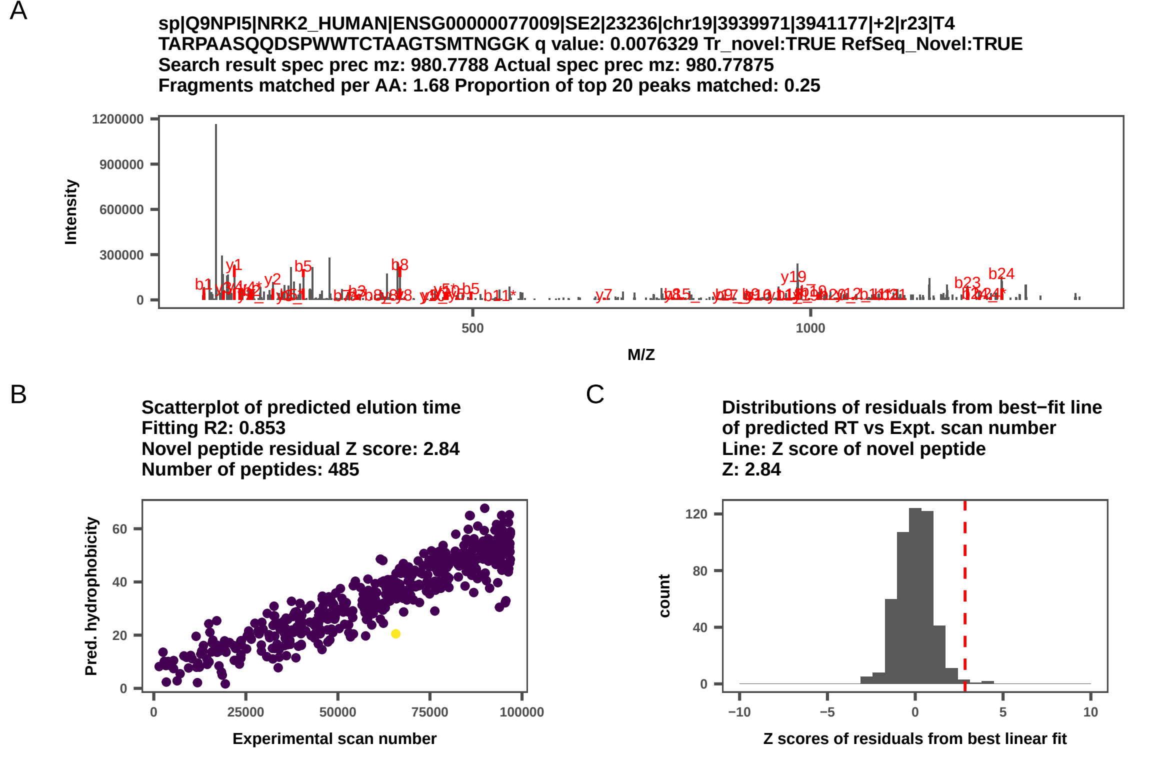
A
sp|Q9NPI5|NRK2_HUMAN|ENSG00000077009|SE2|23236|chr19|3939971|3941177|+2|r23|T4
TARPAASQQDSPWWTCTAAGTSMTNGGK q value: 0.0076329 Tr_novel:TRUE RefSeq_Novel:TRUE
Search result spec prec mz: 980.7788 Actual spec prec mz: 980.77875
Fragments matched per AA: 1.68 Proportion of top 20 peaks matched: 0.25
Intensity
1200000
900000
600000
300000
0
y1
b5
b8
y2
y19
b23
b24
b1
y7
500
1000
M/Z
B
Scatterplot of predicted elution time
Fitting R2: 0.853
Novel peptide residual Z score: 2.84
Number of peptides: 485
Pred. hydrophobicity
60
40
20
0
0
25000
50000
75000
100000
Experimental scan number
C
Distributions of residuals from best−fit line
of predicted RT vs Expt. scan number
Line: Z score of novel peptide
Z: 2.84
count
120
80
40
0
−10
−5
0
5
10
Z scores of residuals from best linear fit
